# Ergot in Chronic Uterine Catarrh

**Published:** 1877-05

**Authors:** 


Ergot in Chronic Uterine Catarrh.—As the operation of ergot given internally is very tedious, and its hypodermic injection is attended with difficulties in private practice, Dr. Goreleitschenko has tried the effect of pencilling the canal of the cervix uteri with solution of ergot (ergotin, 2 scruples; glycerin and distilled water, of each 2 drachms,) leaving the pencil within the canal from one to three minutes. In this way he has treated twenty-nine cases of chronic metritis attended with chronic catarrh. In twenty-two of these the discharge ceased, the swollen state of the mucous membrane disappearing, and the uterus itself being smaller and firmer. The subjective symptoms also ceased, and regular menstruation ensued. In the other seven cases the improvement which ensued was soon followed by relapse, the uterus in these cases having undergone more advanced alterations.—*St. Petersburg Medical Woch.*

